# Changes in the Activities of Antioxidant Enzymes in the Fat Body and Hemolymph of *Apis mellifera* L. Due to Pollen Monodiets

**DOI:** 10.3390/antiox14010069

**Published:** 2025-01-09

**Authors:** Maciej Sylwester Bryś, Krzysztof Olszewski, Maciej Bartoń, Aneta Strachecka

**Affiliations:** 1 Department of Invertebrate Ecophysiology and Experimental Biology, University of Life Sciences in Lublin, 20-950 Lublin, Poland; 2 Subdepartment of Apidology, Institute of Biological Basis of Animal Production, Faculty of Animal Sciences and Bioeconomy, University of Life Sciences in Lublin, 20-950 Lublin, Poland; krzysztof.olszewski@up.lublin.pl; 3 Department of Animal Food Technology, Faculty of Food Science and Biotechnology, University of Life Sciences in Lublin, 20-950 Lublin, Poland; maciej.barton@up.lublin.pl

**Keywords:** antioxidant system, diets, nutrition, immunity, honey bee

## Abstract

The increasing prevalence of monocultures has reduced floral diversity, diminishing pollen diet variety for bees. This study examines the impact of monofloral pollen diets (hazel, rapeseed, pine, buckwheat, *Phacelia*, goldenrod) on the antioxidant enzyme activities in the fat body from tergite 3, tergite 5, sternite, and hemolymph of honey bees. We show that pollen from plants such as rapeseed, *Phacelia*, buckwheat, and goldenrod (rich in phenolic compounds and flavonoids) increases the activities of SOD, CAT, GST, and GPx in the fat body and hemolymph compared to the control group. Although it is commonly known that a monodiet is one of the stress factors for bees, the increase in the activities of these enzymes in the hemolymph and fat body of workers fed with pollen candy compared to those fed only sugar candy has a positive (although inconclusive) effect. These activities in the hemolymph and fat body of bees fed with pollen from anemophilous plants are usually lower compared to those in bees fed with candy containing 10% pollen from rapeseed, *Phacelia*, buckwheat, or goldenrod. Further research is needed to fully understand the complex interactions among monofloral pollen diets, antioxidant enzyme activities, and the overall physiology of honey bees.

## 1. Introduction

The honey bee diet consists of nectar and pollen. Simple sugars present in nectar are utilized by worker bees as a primary energy source, for example for flight and thermoregulation, while pollen provides essential nutrients, such as proteins, fats, and enzymes [1,2]. Access to pollen produced by various plant species positively influences physiological parameters such as worker lifespan, the size of hypopharyngeal glands, and gene expression levels and modulates immune competence, as well as conditioning the energy reserves stored in the fat body [2,3,4,5]. Pollen also contains antioxidant compounds, the concentrations of which vary depending on the plant of origin. Antioxidant compounds found in pollen are synthesized as secondary metabolites [6]. These compounds, known as free radical scavengers, include phenolic acids and flavonoids [7]. In bee pollen, the most common forms of flavonoids are quercetin, flavones, isoflavones, flavanones, anthocyanins, catechins, and isocatechins [8]. Depending on the botanical origin, the composition of phenolic acids present in bee pollen also varies [6]. In the context of humans, regular consumption of bee pollen is of great significance for the prevention of metabolic diseases as well as cardiovascular and neurodegenerative disorders, all of which are closely related to oxidative damage [7,9,10].

In Central Europe, spring plants such as hazel, willow, rapeseed, and blackthorn play a crucial role in the development of bee colonies. In addition to their energetic properties, early spring pollens contain antioxidants. Strong antioxidant properties are characteristic of the pollen of *Brassica napus* and *Prunus mume* [11]. It turns out that monofloral pollen, primarily from *Salix* sp., is characterized by a higher total content of phenolic compounds (TPC) compared to other monoflorals, such as *Cistus* sp., *Taraxacum* sp., Rosaceae, Apiaceae, and even by higher concentrations than multifloral pollen [7,12]. In Poland and throughout Europe, the pollen of summer and early autumn plants, such as buckwheat, *Phacelia*, and goldenrod, is crucial and determines the good wintering of bees. The chemical composition, including protein, lipid, and antioxidant profiles, of the selected bee pollen samples is summarized in Table 1. The chemical composition of pollen is specific to each plant species, while the content of polyphenols may differ between flower pollen and nectar [13,14,15,16]. *Phacelia* pollen has a well-balanced biochemical composition, with an exceptionally high concentration of crude protein (27.44%) and is a good source of phenolics, flavonoids, and other antioxidants [14]. Asteroideae pollen, such as *Solidago* sp., contains 37 different polyphenols, with flavonols and flavonoid glycosides predominating, both of which act as strong free radical scavengers [17]. No information has been found regarding the antioxidant properties of goldenrod pollen, but the total content of polyphenols in samples of goldenrod honey ranges from 1.19 to 6.16% and that of flavonoids from 0.53 to 2.21% [18]. Pine pollen exhibits antioxidant properties attributed to the presence of flavonoids and phenolic acids. These compounds contribute to the scavenging activity of the DPPH radical and hydrogen peroxide [19,20]. Bee pollen not only offers a rich source of nutrients, but also exhibits antioxidant properties, likely attributable to its high content of polyphenols and flavonoids. These compounds are thought to modulate the activity of antioxidant enzymes in the hemolymph and fat body of bees, thereby contributing to their resistance. Unfortunately, it happens that bees collect pollen loads contaminated with pesticides. The antioxidant properties of pollen are weakened or lost due to the presence of pesticides. The active substances found in bee pollen impair bee metabolism or can lead to death [21,22].

In recent times, there has been a significant interest in bee pollen as a “superfood” for humans and other animals due to its nutritional and therapeutic properties. According to a literature review, scientists determine the physicochemical and biological properties of pollen depending on its botanical and geographical origin [23,24,25]. Multifloral pollen loads differ in terms of physicochemical, functional, and sensory properties due to seasonal and regional changes, while the properties of monofloral pollens with a specific botanical origin are fairly consistent [24]. It is generally accepted that the properties of pollen change depending on storage (time, temperature, etc.) due to the lactic acid fermentation process, which increases the availability of nutrients found in the cytoplasm of pollen grains [26]. This hypothesis was contradicted by Caroll et al. [27], who proved that bees prefer fresh pollen. Worker bees fed with pollen stored for 1, 7, and 10 days did not show differences in selected physiological parameters, suggesting that the nutritional value and digestibility of the pollen did not change over time [27].

There is a strong link between the immune system of the honey bee and the quality of pollen food [3,28]. High-quality pollen provides amino acids necessary for the synthesis of immune peptides. The antioxidant system is one of the immune mechanisms [29,30,31]. ROS are generated primarily in mitochondria, which are, among others, present in the fat body of honey bees [32]. Increased production of reactive oxygen species (ROS) in the fat body causes damage to cellular components, the inactivation of enzymes and transport proteins, lipid peroxidation, inflammation, premature aging, etc. [30,33]. Stress factors can increase ROS production. Internal stressors include, for example, age, disease state, parasites [31,34,35,36,37,38], while external (environmental) factors comprise pesticides, landscape changes, monoculture, exposure to electromagnetic fields, as well as the type of beekeeping management and beekeepers’ mistakes such as adulteration of wax with stearin or paraffin [39,40,41,42]. The antioxidants produced by the organism protect it against harmful ROS. In bees, antioxidant enzymes such as superoxide dismutase (SOD), catalase (CAT), and glutathione S-transferase (GST) have been identified. However, thioredoxin reductase (TrxR) and thioredoxin peroxidase (TPX) exhibit a similar activity in bees to these antioxidants [43,44]. We hypothesize that (1) different types of pollen affect the activities of antioxidant enzymes in the fat body and hemolymph in different ways, and that (2) pollen produced by insect-pollinated plants has a greater impact on increasing the activities of antioxidant enzymes than pollen from wind-pollinated plants. Finally, we want to find out whether (3) monodiets can disrupt the activities of the antioxidant enzymes of the fat body and hemolymph and be harmful to worker honey bees. The aim of the study is to determine the effect of pollens from individual plants (hazel, rapeseed, pine, buckwheat, *Phacelia*, goldenrod) on antioxidant parameters (SOD, CAT, TAC, GPx) in the fat body and hemolymph.

**Table 1:** Comparison of total protein, fatty acids, and phenolic and flavonoid compound content of bee pollen samples.

Taxon	Total Protein Content [%]	Fatty Acid Composition	Phenolic and Flavonoid Compounds	Literature
*Corylus* sp.	No literature data available			
*Brassica napus*	20	TS: 47.6 g/100 g TP: 36.7 g/100 g Fat: 6.56 g/100 g	TFC: 2.9–4.9 FRAP: 8.3–9.3 DPPH: 12.8–13.5	[13,14,23]
*Pinus* sp.	10.84	TS: 13.6 mg/g TP: 13.2 mg/g Fat: 7.3 g/100g	TFC: 2–6 mg/g TPC: 1.9–3.5 mg/kg	[19]
*Phacelia* sp.	27.44	Fat: 5.35%	TPC: 27.5 mgGAE/g TFC: 3.58 mgQE/g FRAP: 8.16 mgAAE/g DPPH: 10.39 mgTE/g	[13,14]
*Fagopyrum* sp.	11.4–14.3	TS: 37.2 g/100 g TP: 21.6 g/100 g Fat: 5.15 g/100 g	TFC: 0.24 ppm,	[16,23]
*Solidago* sp.	>20	No literature data available		[45]

Total saturated fatty acids (TS); total polyunsaturated fatty acids (TP); total phenolic content (TPC); total flavonoid content (TFC); antioxidant capacity-FRAP, antioxidant capacity-DPPH.

## 2. Materials and Methods

### 2.1. Selection of Pollen Loads, Palynological Analyses, and Preparation of Pollen Monodiets

**Figure 1:**
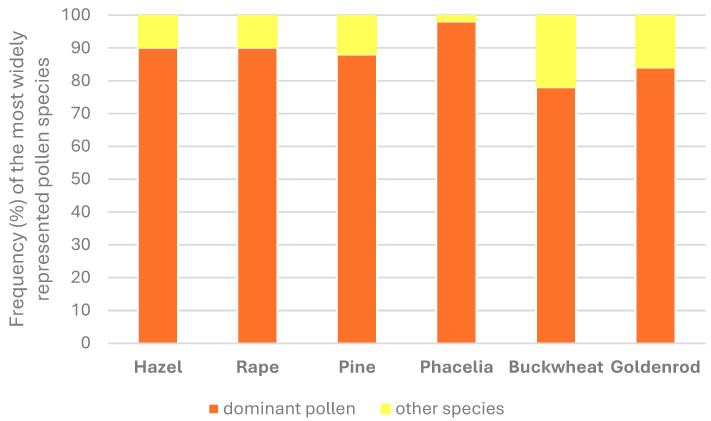
Pollen frequency in the examined pollen loads.

The selection of monofloral pollen diets was dictated by the common occurrence of plant pollen in a temperate climate. Fresh pollen was collected using pollen traps mounted in front of hives belonging to beekeepers from the Lubelskie and Podkarpackie provinces (Poland). The pollen was manually sorted by color to obtain the dominant pollen from a specific plant in each sample [46]. To confirm the botanical origin, microscopic preparations were made and analyzed using the methods described by Filipiak et al. [47]. Microscopic pollen preparations were then used to confirm the identity of the pollen through morphological features. Samples were examined using a microscope MBL 800 at a 40 × 15 magnification. At least 300 pollen grains were counted per slide, with two replicate counts for each slide. Pollen assignation was performed to the most accurate taxon (species, genus). Pollen load samples were obtained from wind-pollinated plants, i.e., hazel (*Corylus* sp.) and pine (*Pinus sylvestris* L.), and insect-pollinated plants, i.e., rapeseed (*Brassica napus* L.), *Phacelia* (*Phacelia tanacetifolia* Benth), buckwheat (*Fagopyrum esculentum* Moench), and goldenrod (*Solidago* sp.). The percentage of the dominant pollen in the sample is presented in Figure 1.

**Figure 2:**
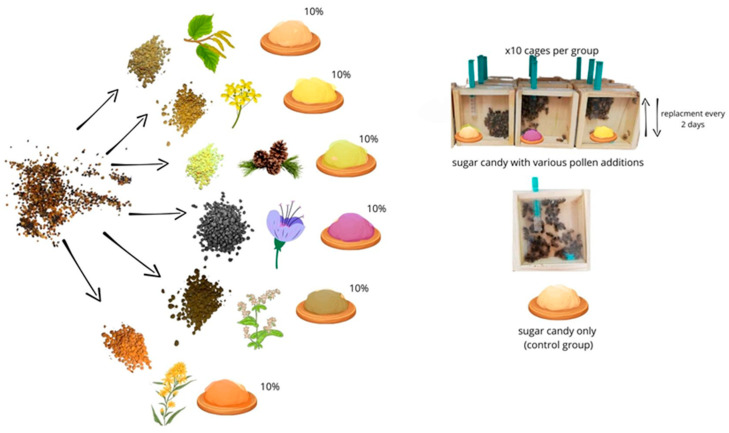
Sugar candy preparation and serving scheme for various pollen additions.

The sorted pollen was frozen at −25 °C until the preparation of sugar candy with the respective pollen types. Commercial sugar candy (Apifonda, Łysoń, Poland) was divided into seven equal parts. The first part did not contain any pollen supplement (bees from the control group were fed with it). To the remaining parts of sugar candy, one of the six types of pollen, previously micronized using an electric mill, was added at a concentration of 10% (Figure 2). The addition of 10% pollen in this experiment was valid economically, confirmed by its effectiveness [4], and was also based on the theoretical protein requirement of bees at an approximate ratio of 10% protein and 90% carbohydrates according to Altaye [48]. The manually mixed candy batches were frozen at −25 °C and gradually thawed before feeding the bees.

### 2.2. Analyses of Active Substance Residues in Pollen Loads

The pollen samples were analyzed for pesticide residues. The initial sample preparation was based on the QuEChERS method by Anastassiades et al. [49]. A 2 g portion of pollen was weighed and homogenized. The prepared pollen was subjected to salting out and solid–liquid extraction using acetonitrile [50]. For pesticide detection, liquid chromatography coupled with mass spectrometry (LC-MS) (Agilent 6470 Triple Quad) and gas chromatography mass spectrometry (GC-MS) (Agilent 7000 Triple Quad) analyses were performed according to the methodology described by Kaila [50], with a limit of quantification (LOQ) of 0.01 mg kg^−1^. To detect polar pesticides, 2 g of monofloral pollen was homogenized and 10 mL of pure water and 10 mL of cold methanol with 1% formic acid were added. For analysis, 100 μL of standards (depending on the type) or deuterated pesticide analogs were used. The sample was then shaken for 5 min and centrifuged for 5 min. A total of 200 μL of the supernatant was collected and 800 μL of mobile phase A for the chromatograph was added for the analysis of polar pesticides. The mixture was mixed using a vortex. A cellulose filter was applied for filtration according to the QuPPe method. The samples were analyzed using liquid chromatography with mass spectrometry detection (Ultivo Triple Quad). The results are presented in Table 2.

### 2.3. Obtaining One-Day-Old Bees and the Experiment

Queen bees from three hives originating from an apiary belonging to the University of Life Sciences in Lublin (51°22′ N, 22°63′ E), Poland, were placed in an isolator on a single frame to obtain eggs and then larvae of a similar age. After 24 h, the queen was released, and the frame with the brood was left in the hive. After 20 days from egg laying, the frame with the brood was removed from the hive and placed in an incubator (35 °C) until the workers emerged. Then, one-day-old workers from the three hives were mixed to ensure uniformity and placed in 70 sterile wooden cages measuring 12 × 12 × 4 cm with a glass window. The experiment was conducted under controlled conditions with a constant temperature of 32 °C and humidity of 65%. The cages were randomly divided into seven groups (10 cages per group). Each cage contained 40 worker bees. At the beginning of the experiment, one-day-old bees were collected for biochemical analyses. The control group received only sugar candy, while the remaining groups received sugar candy and a 10% addition of one of the specific pollens such as hazel, rapeseed, pine, *Phacelia*, buckwheat, or goldenrod. The pollen diets were introduced from the first day of life and were provided *ad libitum* throughout the experiment. Apart from supplying sugar candy with pollen, water was changed every two days and dead individuals were removed from the cages. At the age of 7 and 14 days, living worker bees were collected from the cages for laboratory analyses (one bee per cage, *n* = 10 bees per group). This resulted in the following dataset: 10 1-day-old workers + 10 worker bees × 7 groups × 2 samplings.

### 2.4. Laboratory Analyses

#### 2.4.1. Obtaining Biological Material for Research

Hemolymph and fat body were used as materials for biochemical analyses [4]. The method of collecting hemolymph from the venous sinus in the bee’s abdomen was validated by Łoś and Strachecka [51]. After collecting hemolymph from each bee individually, it was placed in a separate Eppendorf tube containing 200 μL of 0.6% NaCl. The collected samples were immediately frozen at −25 °C for further analysis. After collecting the hemolymph, the bees were frozen. Subsequently, the individuals were thawed gradually and the fat body from the third and fifth tergites and the sternite was prepared according to the methodology described by Bryś et al. [4]. The choice of these three locations for biochemical analyses was based on previous studies by Strachecka et al. [52] which demonstrated that the fat body from these locations is, metabolically, the most active. Next, the tissues were manually homogenized and centrifuged at 4 °C for 1 min at 3000× *g*. The supernatants were frozen at −25 °C for further biochemical analyses.

#### 2.4.2. Biochemical Analyses

The following parameters were determined in hemolymph solutions and fat body supernatants:

- Superoxide dismutase (SOD) activity according to the method described in the commercial kit SOD Assay Kit, Sigma Aldrich, Schnelldorf, Germany, no. 19160-1KT-F;
- Glutathione S-transferase (GST) activity according to the method described in the commercial kit Glutathione S-transferase Assay Kit, Sigma Aldrich, Schnelldorf, Germany, no. MAK 435-1KT;
- Glutathione peroxidase (GPx) activity according to the method described in the commercial kit Glutathione Peroxidase Assay Kit, Sigma Aldrich, Schnelldorf, Germany, no. MAK437-1KT;
- Catalase (CAT) activity according to the method described in the commercial kit Catalase Assay Kit, Cayman Chemical Company, East Ellsworth Road Ann Arbor, USA, Item: 707002;
- Total antioxidant capacity (TAC) according to the method described in the commercial kit Antioxidant Assay Kit, Cayman Chemical Company, East Ellsworth Road Ann Arbor, USA, Item: 709001.

The antioxidant enzyme activities were calculated per 1 mg of protein.

### 2.5. Statistical Analyses

Statistical analyses were performed using Statistica formulas (TIBCO Software, Palo Alto, CA, USA) 13.3 (2017), version for Windows—StatSoft Inc., Tulsa, OK, USA. Data distribution was checked using the Shapiro–Wilk test. The effect of the tissue/location of fat body (hemolymph and fat body from tergite 3, tergite 5, and sternite) in the 1-day-old workers (*n*  =  10 bees) on SOD, GST, GPx, and CAT activities, and TAC levels were measured. ANOVA was used for normally distributed data and the Kruskal–Wallis test was employed for non-normally distributed data. Activity’s tissues/locations of fat body for the enzymes (SOD, GST, GPx, CAT, and TAC) for normally distributed data were compared with the Tukey HDS test, and, for non-normally distributed data, the Mann–Whitney U test was used. For each type of pollen, the effect of the tissue/location of fat body (hemolymph and fat body from tergite 3, tergite 5, sternite) on SOD, GST, GPx, and CAT activities, and TAC levels was assessed in the 7- and 14-day-old workers (*n* = 10 bees per group). ANOVA was used for normally distributed data and the Kruskal–Wallis test was employed for non-normally distributed data. SOD, GST, GPx, CAT activities, and TAC levels for hazel, rape, pine, *Phacelia*, buckwheat, and goldenrod pollen were compared with respect to the tissue/location of fat body (hemolymph and fat body from tergite 3, tergite 5, and sternite). The student’s *t*-test was used for normally distributed data and the Mann–Whitney U test was employed for non-normally distributed data. Likewise, the influence of pollen from wind-pollinated plants (hazel and pine) on enzyme activities was compared with that of pollen from insect-pollinated plants (rapeseed, *Phacelia*, buckwheat, goldenrod).

## 3. Results

### 3.1. Activities of Superoxide Dismutase (SOD), Glutathione S-Transferase (GST), Glutathione Peroxidase (GPx), Catalase (CAT), and Total Antioxidant Capacity (TAC) Levels in One-Day-Old Workers

**Figure 3:**
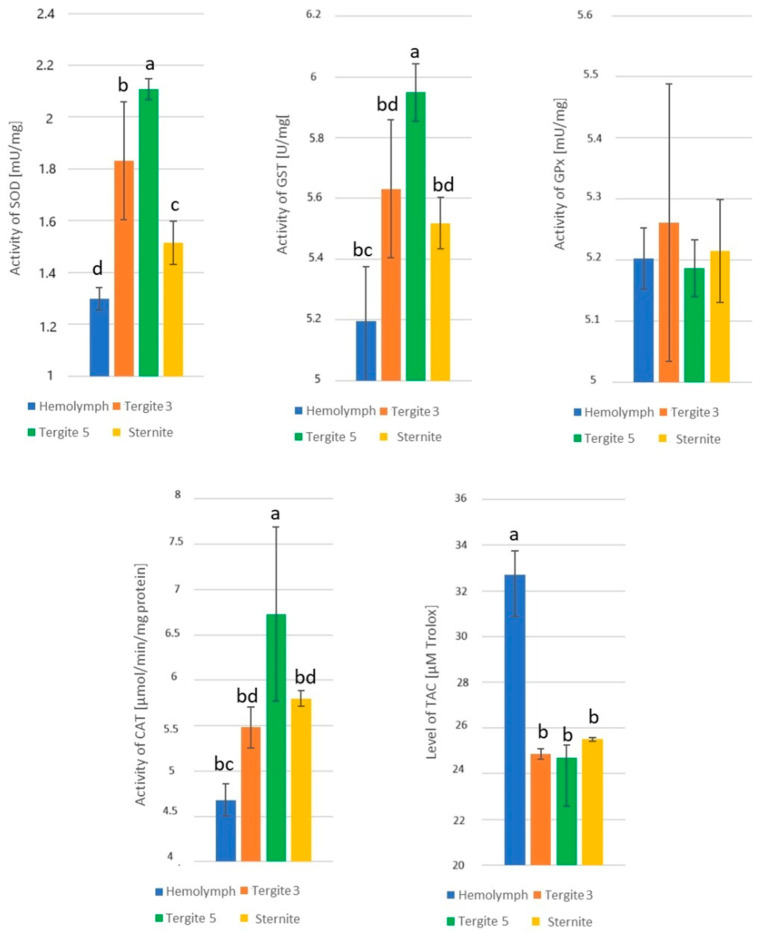
Superoxide dismutase (SOD), glutathione S-transferase (GST), glutathione peroxidase (GPx), and catalase (CAT) activities and total antioxidant capacity (TAC) levels in the hemolymph and fat body from tergite 3, tergite 5, and sternite in 1-day-old workers; a,b,c,d—differences between tissues/location for individual enzymes significant at *p* ≤ 0.01; *n* = 10; vertical bars indicate standard deviation.

The effect of the tissue/location of fat body (hemolymph and fat body from tergite 3, tergite 5, and sternite) on the enzyme activities was statistically significant: SOD − H = 36.63, *p* = 0.000; GST − F = 85.12, *p* = 0.000; GPx − H = 7.09, *p* = 0.069; CAT − F = 70.69, *p* = 0.000; and TAC − H = 24.84, *p* = 0.000, respectively. The highest activities of SOD, GST, and CAT were identified in tergite 5 and the lowest in the hemolymph (Figure 3). There were no statistically significant differences in GPx activities, whereas TAC levels in the hemolymph were statistically significantly higher than those in all the fat body locations, which, in turn, did not significantly differ among each other (*p* ≤ 0.05).

### 3.2. The Effect of the Tissue and Location of the Fat Body on the Enzyme Activities

In all the pollen monodiets and in the control group, both in 7-day-old and 14-day-old workers, the location of the fat body exerted a significant effect on the activities of SOD, GST, GPx, and CAT enzymes and on the TAC levels (Table 3).

### 3.3. Activities of Superoxide Dismutase

**Figure 4:**
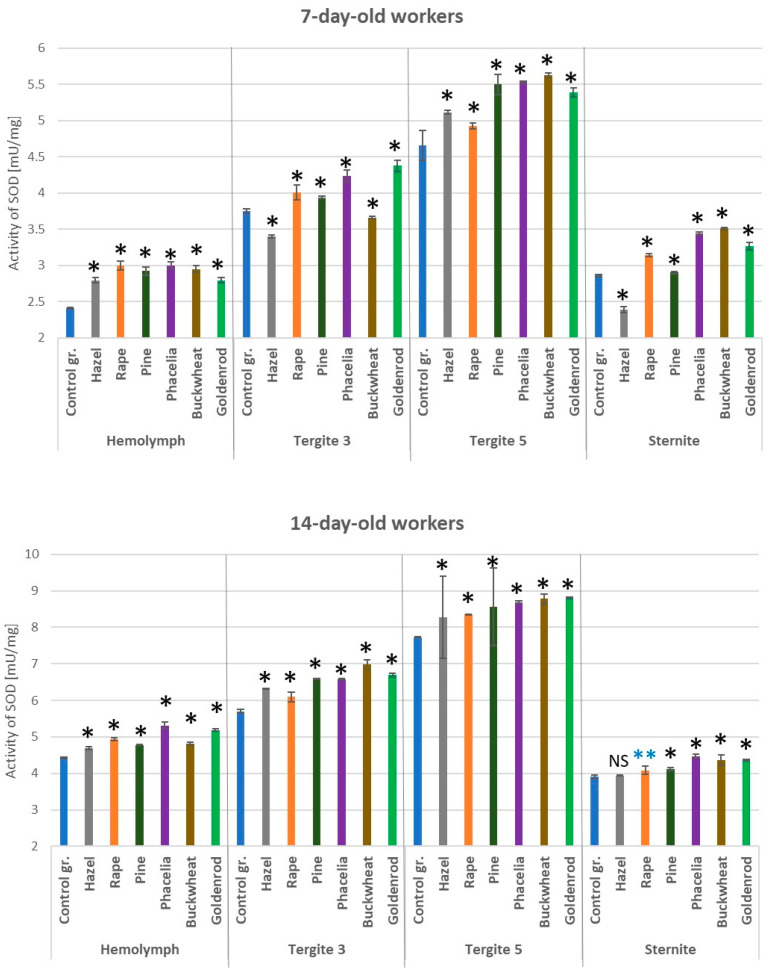
Superoxide dismutase (SOD) activities in the hemolymph and fat body from tergite 3, tergite 5, and sternite in the 7- and 14-day-old workers fed sugar candy only and in those fed sugar candy with various pollen additions; *—differences between the pollen-fed workers and the control group workers in the same tissues/locations are significant at *p* = 0.01; **—*p* = 0.05; *n* = 10; NS—not significant; vertical bars indicate standard deviation.

In both the 7- and 14-day-old workers fed with a 10% pollen supplement (except for hazel and buckwheat in the tergite 3 and hazel in the sternite), the SOD activities were statistically significantly higher compared to those in the control group (Figure 4). Compared to the other locations, the highest values of superoxide dismutase were observed in the tergite 5. In all cases (tissues/locations of fat body), the SOD activities were higher in the 14-day-old than in the 7-day-old workers. The SOD activities in the hemolymph and fat body of the 7- and 14-day-old workers fed with hazel pollen candy were statistically significantly higher compared to the bees fed with candy containing different pollens produced by insect-pollinated plants (*p* = 0.01).

### 3.4. Activities of Glutathione S-Transferase

**Figure 5:**
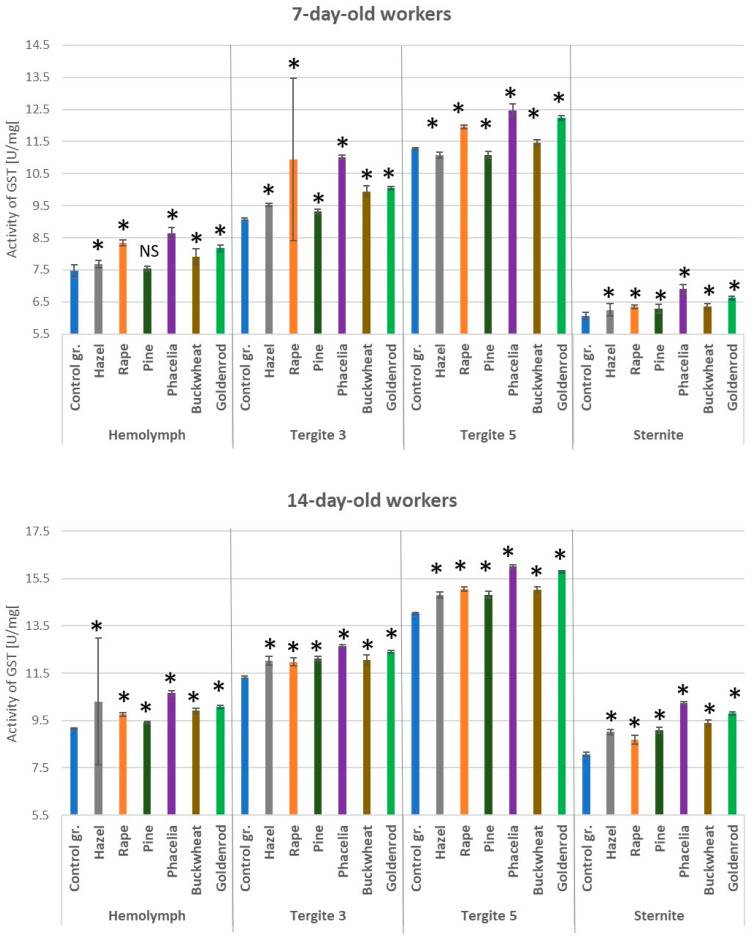
Glutathione S-transferase (GST) activities in the hemolymph and fat body from tergite 3, tergite 5, and sternite in the 7- and 14-day-old workers fed sugar candy only and in those fed sugar candy with various pollen additions; *—differences between the pollen-fed workers and the control group workers in the same tissues/locations are significant at *p* = 0.01; *n* = 10; NS—not significant; vertical bars indicate standard deviation.

The addition of pollen to the sugar candy diet increased GST activities in both the hemolymph and the fat body in 7- and 14-day-old workers (Figure 5). Statistically significantly lower GST activities were found in the sternite compared to the other locations. In the bees fed only with sugar candy, especially in the 14-day-old ones, GST activities were lower than in those fed with candy containing individual pollen additions. The highest GST activities in the hemolymph and individual locations of the fat body were observed in the bees fed sugar candy containing *Phacelia* pollen. The GST activities in the hemolymph, tergite 3, tergite 5, and sternite of the bees at 7 days of age fed sugar candy with hazel or pine pollen were statistically significantly lower compared to those in the workers fed with pollen produced by insect-pollinated plants (*p* = 0.01).

### 3.5. Activities of Glutathione Peroxidase

**Figure 6:**
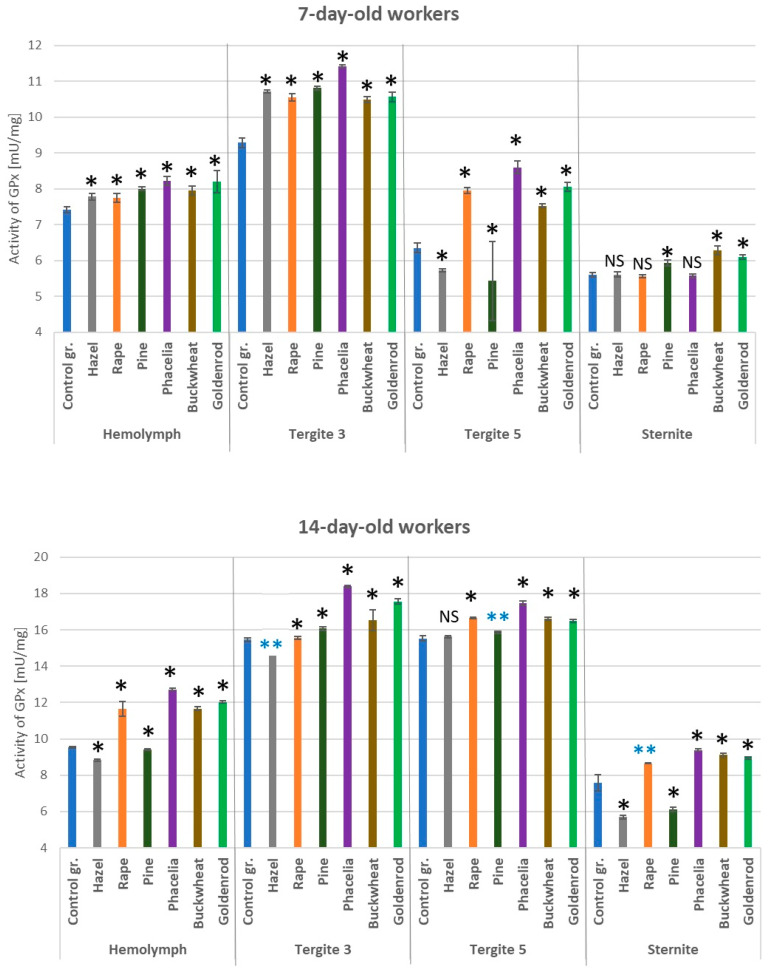
Glutathione peroxidase (GPx) activities in the hemolymph and fat body from tergite 3, tergite 5, and sternite in the 7- and 14-day-old workers fed sugar candy only and in those fed sugar candy with various pollen additions; *—differences between the pollen-fed workers and the control group workers in the same tissues/locations are significant at *p* = 0.01; **—*p* = 0.05; *n* = 10; NS—not significant; vertical bars indicate standard deviation.

The addition of pollen to sugar candy caused an increase in GPx activities in the hemolymph and respective fat body locations in the 7- and 14-day-old workers (Figure 6). Particularly high GPx activities were observed in tergite 3 on the 7th day of life of the bees and in tergites 3 and 5 on the 14th day. Glutathione peroxidase was always higher in the workers which consumed *Phacelia* pollen (sugar candy + 10% pollen) in tergite 3 compared to those fed sugar candy only. The GPx activities in the hemolymph and fat body of the bees fed pollen from wind-pollinated plants were statistically significantly lower only in the 14-day-old workers compared to the bees fed sugar candy with 10% pollen from rape, *Phacelia*, buckwheat, or goldenrod (*p* = 0.01).

### 3.6. Activities of Catalase

**Figure 7:**
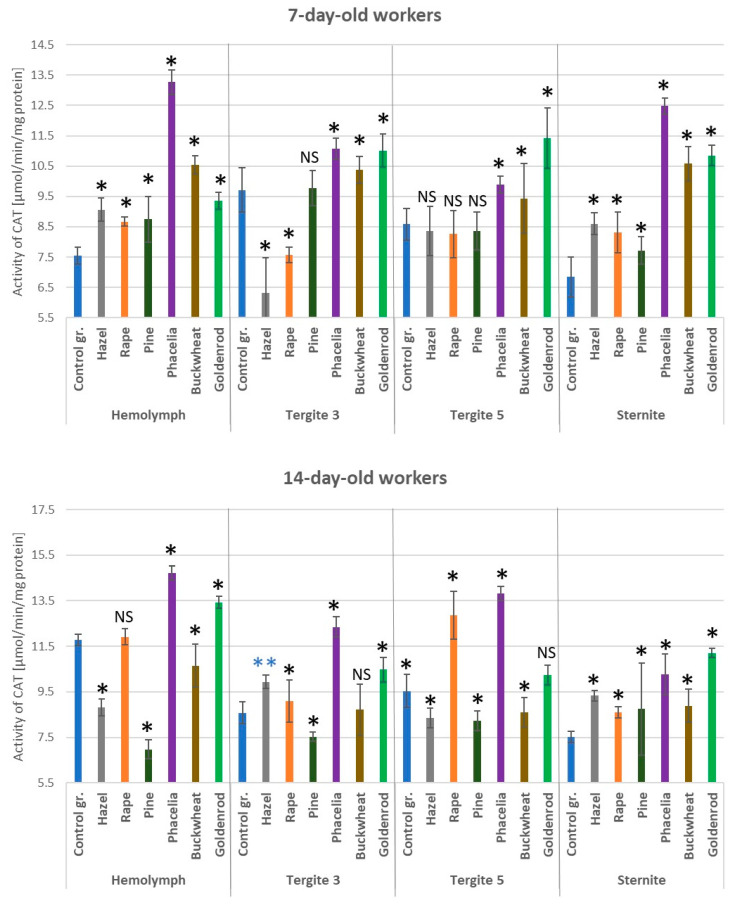
Catalase (CAT) activities in the hemolymph and fat body from tergite 3, tergite 5, and sternite in the 7- and 14-day-old workers fed sugar candy only and in those fed sugar candy containing various pollen additions; *—differences between the pollen-fed workers and the control group workers in the same tissues/locations are significant at *p* = 0.01; **—*p* = 0.05; *n* = 10; NS—not significant; vertical bars indicate standard deviation.

Catalase activities were higher in the hemolymph of the 7-day-old workers and in the sternite fat body of the 7- and 14-day-old workers fed candy with pollen (hazel, rapeseed, pine, *Phacelia*, buckwheat, and goldenrod) compared to those fed with sugar candy alone (control group) (Figure 7). A similar situation was observed in the fat body of tergite 3 and 5 in the 7-day-old bees fed *Phacelia*, buckwheat, and goldenrod pollen. In the 14-day-old workers fed candy containing pine pollen, lower CAT activities were observed in the hemolymph and fat body of tergites 3 and 5 compared to the control group. Considering catalase activities, the lowest statistically significant differences (*p* < 0.05) were observed in the bees fed with hazel and pine pollen compared to the other groups of bees fed with sugar candy containing a 10% addition of pollen from entomophilous plants.

### 3.7. Levels of Total Antioxidant Capacity

**Figure 8:**
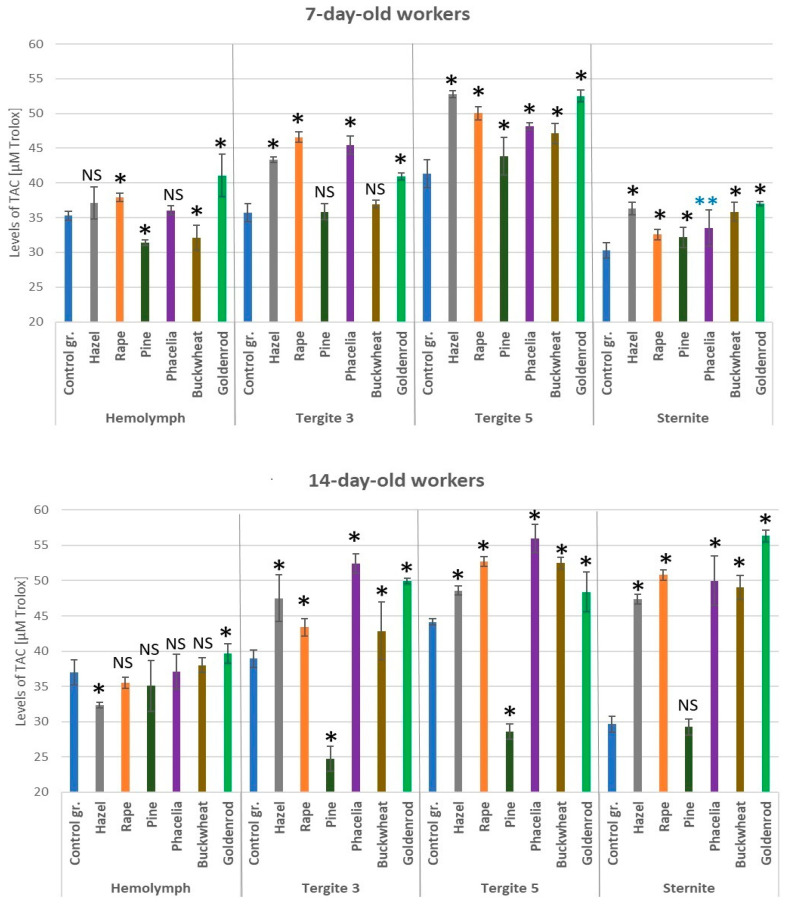
Total antioxidant capacity (TAC) levels in the hemolymph and fat body from tergite 3, tergite 5, and sternite in the 7- and 14-day-old workers fed sugar candy only and in those fed sugar candy containing various pollen additions; *—differences between the pollen-fed workers and the control group workers in the same tissues/locations are significant at *p* = 0.01; **—*p* = 0.05; *n* = 10; NS—not significant; vertical bars indicate standard deviation.

TAC levels were higher in the fat body of tergites 3 and 5 in the 7-day-old bees fed candy with pollen supplements compared to those fed sugar cake alone (Figure 8). In the 14-day-old workers, higher TAC levels were observed in the fat body of sternites and tergites 3 and 5 after feeding the bees with pollen-enriched candy (except pine pollen) compared to those in the control group. The TAC levels were statistically significantly higher in the fat body of tergite 5 and sternite of the 7-day-old workers fed candy with hazel pollen than in those of bees fed with other types of pollen (*p* < 0.05).

## 4. Discussion

The activities of antioxidant enzymes such as SOD, CAT, GST, GPx, and TAC in bees are commonly used to monitor oxidative stress. To assess antioxidant activities, homogenates from entire abdomens [37,53,54,55] and hemolymph [29,30,39,40,41,56] are widely used. There are few publications on the activities of these enzymes in the fat body. Santos et al. [32] reported the expression of antioxidant genes (MnSOD, CuZnSOD, catalase, Gst1, and GSH/GSSG) in the fat body of queen and worker larvae. Brajcha et al. [57] compared the expression of genes (including antioxidant ones) of fat body cells in both long-lived winter and short-lived summer worker bees (the youngest stage of hive bees and forager bees). Hsu and Hsieh [58] characterized the activities of CAT, GPx, and SOD in trophocytes and other fat body cells in 1- and 50-day-old workers. In our previous study [31], we focused on comparing the activities of antioxidants in different locations of the fat body in different castes of bees—queens, workers, and rebels just after their emergence. This publication supplements the knowledge of the fat body physiology with the following content: (1) it characterizes the activities of antioxidants in different segments/locations of the fat body not only in 1-day-old workers, but also in nest workers at the age of 7 and 14 days, (2) it presents the influence of particular pollens (in a monodiet) on the activities of the above enzymes in the fat body of the sternite, tergite 3, and tergite 5 and in the hemolymph of workers up to 14 days of age, and (3) it compares the influence of pollen from anemophilous and entomophilous plants on the antioxidant system in two tissues crucial for bee immunity.

The underlying problem of beekeeping all over the world, and especially in Europe, is an improper, poorly balanced pollen diet which results in reduced immunity [3,59,60]. Indeed, one of the lines of defense is the antioxidant system. We assessed the effect of specific pollens on the physiological/biochemical parameters of the fat body and hemolymph in laboratory conditions (in cage tests), with strictly controlled parameters and limited environmental influence. Filipiak et al. [46] showed that even a small addition of pollen to the diet of bees has a beneficial effect on their vitality. In turn, Jachuła et al. [61] found that there is no ideal mixture that will meet all the metabolic needs of bees. In order to verify the dietary potential of pollen, it is necessary to know its content of polyphenols and flavonoids, the composition, as well as their direct effect on the bee’s organism. It is undeniable that a multipollen diet is optimal for honey bees, but to fully grasp how the properties of pollens interact with honey bee antioxidant system, we need to examine this diet through the lens of monodiets. Hence, our research complements this discourse.

The addition of hazel, pine, rapeseed, *Phacelia*, buckwheat, and goldenrod pollen to sugar candy increased the activities of SOD and GST in both the hemolymph and the fat body of the sternite, tergite 3, and tergite 5 in the workers on the 7th and 14th day of their life. Similar trends were observed in the case of GPx activities in the hemolymph and fat body of tergite 3 in the 7- and 14-day-old workers and, additionally, in the fat body of tergite 5 in the 14-day-old bees. Moreover, we observed an increase in the activities of these three enzymes corresponding with the age of the bees. In the case of CAT, the pollen diet resulted in higher activities in the hemolymph and fat body of tergite 3 (exception: hazel and pine), tergite 5, and sternite in the 7-day-old workers. In the remaining cases, the trends were not so clear. Hsu and Hsieh [58] indicate that CAT activities in the fat body increase with age, while SOD activities decrease. The difference between our results and those obtained by these authors may be due to the fact that we presented the activities of the antioxidants in individual segments/locations of the subcuticular fat body. Hsu and Hsieh [58] did not specify the part of the fat body sampled (visceral or subcuticular) or its location (segment). In our study, we confirmed the observations of Strachecka et al. [31] that antioxidant enzyme activities vary depending on the type of tissue (hemolymph vs. fat body) and fat body segments/locations (tergite 3, tergite 5, and sternite). These authors showed that the activities of SOD and CAT were always highest in the sternite, while the levels of TAC were highest in tergite 3 in the workers. In our study, a monodiet produced the lowest activities of SOD, GST, and GPx in the sternite (with some exceptions) in each group of bees. The activities of these enzymes are closely related to the functioning/metabolism of individual organelles in the fat body cells and ROS neutralization reactions. As suggested by Scofied and Amdam [62], nurse bees maintain high levels of lipids and other substances in the abdomen, including the fat body, while foragers have very low levels of these compounds, a phenomenon which probably contributes to the efficient performance of their social role and, thus, to the colony’s fitness. This results from the evolution of eusocial organisms [62], but also from the adaptation to changing environmental conditions [63], as indicated also in our studies showing how much influence a monodiet has on the bee organism. Although the monodiet is a stress factor for bees, by comparing the hemolymph and fat body of bees fed sugar candy with pollen, antioxidant activities were usually found to be higher than in the control group. Similar conclusions were also formulated by Yazlovytska et al. [53]. Moreover, under cage conditions, these authors showed that bees which received a sucrose solution with the addition of willow pollen or artificial rapeseed beebread or artificial willow beebread lived longer and were characterized by higher values of lipid peroxidation and catalase activities (as oxidative stress biomarkers) in their heads and abdomen compared to the group fed with the sucrose solution only. These CAT activities are consistent with our results, especially in reference to the 7-day-old bees. The authors of that publication, unfortunately, did not state how they suspended/mixed the pollen in the sugar solution and how they fed the suspension to the bees, e.g., whether it was an even feeding. We avoided this mistake by giving the bees pollen in sugar candy.

TAC levels were the least consistent and there were virtually no statistical differences in the hemolymph of the 14-day-old bees, regardless of the diet. This is probably due to the fact that the total antioxidant activity is the sum of the activities of enzymatic and non-enzymatic antioxidants. Nevertheless, it was noted that the TAC level increased with age in tergite 3 and sternite in bees fed with a 10% addition of rapeseed, *Phacelia*, buckwheat, or goldenrod. Based on literature data, the pollen of rapeseed, *Phacelia*, and buckwheat plants has a high content of phenolic acids and flavonoids [16,64]. Flavonoids and carotenoids are considered non-enzymatic antioxidants. Carotenoids protect the lipoproteins found, for example, in the fat body from singlet oxygen, which causes lipid peroxidation [65]. Upon oxidation, flavonoids act as electron donors to molecular oxygen, forming superoxide radicals [66]. There was no information to be found on phenolic compounds in goldenrod pollen, but the concentrations of the main phenolic compounds differ in the inflorescences of *Canadian goldenrod* L. and *Solidago gigantea* Aiton [67]. Since there are differences in the composition of antioxidant compounds in plant extracts, there will most likely also be differences in the composition of pollen. However, the question arises: will pollen produced by different species of goldenrod have the same or different effects on the physiological parameters of hemolymph and fat body? Goldenrod is a key plant species throughout Europe, as it provides vast amounts of pollen (about 100–200 kg of pollen per hectare) for bees. From a botanical point of view, goldenrod is an invasive plant, but, on the other hand, it is valuable for pollinators [68,69]. The industrialization and modernization of agriculture and forestry promote the use of various groups of chemical compounds, including insecticides, herbicides, fungicides, and other pesticides [70,71]. There is increasing research on the toxicity of pesticides to honey bees. It has been observed that contact of bees with pesticides induces oxidative stress [71], and the final effect may be different, even within the same chemical group, and may vary depending on the dose, mode of administration, exposure time, and type of biological sample taken for biochemical determinations [72]. To exclude the influence of pesticides contained in pollen, we determined their concentrations using chromatographic methods. The concentrations of active substances detected in the pollen samples were not a cause for concern, except for rapeseed pollen. Three groups of chemical compounds were identified in rapeseed pollen. Due to the multihectare cultivation of rapeseed practically all over the world, pesticides are used to protect plants, and residues of active substances in rapeseed pollen, beebread, and honey pose a global problem [73]. In our study, the enzymatic activities of antioxidants in the hemolymph of bees fed with candy supplemented with rapeseed pollen were significantly higher (*p* = 0.01) than in the control group. In our case, the active substances detected in rapeseed pollen loads did not have a negative impact on the activities of antioxidant enzymes. Many of the aforementioned stressors have a harmful effect on the homeostasis of the antioxidant system. To counteract the negative effects caused by stress, scientists recommend the use of natural chemical compounds. A positive effect of biostimulants such as curcumin, coenzyme Q10, caffeine, cannabidiol, and vitamin C has been observed, consisting of increasing the activity of the antioxidant enzyme system [29,30,44,74,75,76,77]. There is a high probability that a well-balanced pollen diet will condition an increase in the activities of enzymatic and non-enzymatic markers of oxidative stress. Higher concentrations of, for example, SOD, CAT, and GPx generated by a pollen diet will constitute a kind of barrier against other harmful factors such as varroa, *Vairimorpha*/*Nosema*, etc.

Each pollen type possesses unique biological characteristics that impact various aspects of apian physiology, including its immune and antioxidant systems. This influence is evident in the activities of antioxidant enzymes such as SOD, CAT, GST, GPx, and in the total antioxidant capacity (TAC) levels. However, pollen from insect-pollinated plants induced a greater increase in antioxidant parameters compared to pollen from wind-pollinated plants. Hazel and pine pollen, specifically, led to an increase in SOD and GST activities at 14 days of age compared to the bees fed sugar candy only. Although hazel is a wind-pollinated plant, it is very frequently visited by bees. As one of the first spring pollen sources in Central Europe, it can be crucial for protecting bees from oxidative stress caused by various factors. As Romanovskaja et al. [78] report, shifts in earlier phenological phases during blooming, including in *Corylus* sp., reflect climate change. Wind-pollinated plants prove to be a good way to fill food gaps for bees. Beekeepers frequently sow *Phacelia* between June and July/August to provide bees with a consistent supply of pollen and nectar. This plant’s rapid growth and flowering make it ideal for agricultural areas, offering protein-rich pollen, especially when bee food sources are scarce. Our studies have revealed that *Phacelia* pollen has a pronounced effect on enzyme activities in comparison with hazel, rape, pine, buckwheat, and goldenrod.

Enzyme SOD, GPx, GST, and CAT activities and TAC levels varied among tergite 3, tergite 5, and the sternite. Our earlier research indicates that monodiets influence the concentrations of energy storage compounds in the fat body differently based on the segment [4]. This study, thus, corroborates the segmented organization of the fat body, as proposed by Strachecka et al. [52]. The segmented structure of the fat body underlies the varying activities of antioxidant enzymes (particularly in tergite 5) and energy compounds (in tergite 3 and the sternite), implying distinct physiological functions of individual fat body segments. Diverse stressors disrupt antioxidant activities within bee tissues, notably in the hemolymph. Our study suggests that higher enzymatic activities of CAT, SOD, GPx, and GST and higher levels of TAC in bees fed various pollen monodiets are a consequential response to the presence of certain compounds in the flower pollen. We posit that this enhanced enzymatic activity functions as an endogenous protective mechanism, safeguarding against the deleterious effects of additional stressors, such as parasites.

## 5. Conclusions

Each pollen has individual biological properties, which translate into the functioning of the bee’s organism, including its immune and antioxidant systems. Specific pollen types, such as those from rapeseed, *Phacelia*, buckwheat, and goldenrod, can positively influence the activity of antioxidant enzymes as opposed to sugar candy only, contributing to improved bee health and colony survival. SOD, GPx, and GST activities increased with the age of the bees, while CAT showed the opposite trend, and this parameter may be an indicator of monodiet harmfulness. We provide further evidence that the spring pollen diet plays a significant role in the early development of the short-lived generation of bees. Moreover, summer and autumn pollens, e.g., from *Phacelia*, buckwheat, and goldenrod, support antioxidant systems, creating potential for the long-lived generation. Furthermore, based on literature data, considering the strong antioxidant properties of *Phacelia* pollen, as well as its beneficial effects on antioxidant properties, the introduction of this plant to flower mixtures may have an impact on antioxidant parameters and may counteract unfavorable environmental factors.

## Figures and Tables

**Table 2:** Concentrations of active substances detected in pollen loads, LQ < 0.01 mg kg^−1^.

No.	Pollen Load Samples	Active Substance	Mean ± SD [mg/kg]
1.	Hazel	Anthraquinone	0.053 ± 0.027
2.	Rape	Acetamiprid	0.025 ± 0.013
		Azoxystrobin	0.15 ± 0.07
		Thiminamethoxam	0.009 ± 0.004
3.	Pine	<LOQ	
4.	*Phacelia*	<LOQ	
5.	Buckwheat	<LOQ	
6.	Goldenrod	<LOQ	

Limit of quantification (LOQ) of 0.01 mg kg^−1^.

**Table 3:** Effect of the hemolymph and fat body location from tergite 3, tergite 5, and sternite in different age groups of workers (7 and 14 days) on SOD, GST, GPx, CAT, and TAC activities in *A*. *mellifera* workers fed sugar candy only (control group) and in those fed sugar candy with a pollen addition.

Groups	Age of Workers									
	7-Day-Old					14-Day-Old				
	SOD	GST	GPx	CAT	TAC	SOD	GST	GPx	CAT	TAC
Control gr.	H = 36.67 *p* = 0.000	F = 416.70 *p* = 0.000	F = 222.34 *p* = 0.000	F = 46.81 *p* = 0.000	F = 110.42 *p* = 0.000	H = 36.73 *p* = 0.000	H = 36.73 *p* = 0.000	H = 33.04 *p* = 0.000	F = 153.71 *p* = 0.000	F = 227.25 *p* = 0.000
Hazel	H = 36.64 *p* = 0.000	H = 36.65 *p* = 0.000	H = 35.52 *p* = 0.000	H = 22.09 *p* = 0.000	H = 34.91 *p* = 0.000	H = 36.72 *p* = 0.000	H = 32.69 *p* = 0.000	H = 34.12 *p* = 0.000	F = 15.05 *p* = 0.000	H = 33.61 *p* = 0.000
Rape	H = 36.61 *p* = 0.000	H = 35.35 *p* = 0.000	H = 34.96 *p* = 0.000	H = 20.29 *p* = 0.000	H = 36.63 *p* = 0.000	H = 36.64 *p* = 0.000	H = 36.65 *p* = 0.000	H = 36.66 *p* = 0.000	H = 32.51 *p* = 0.000	H = 36.21 *p* = 0.000
Pine	H = 33.76 *p* = 0.000	H = 36.65 *p* = 0.000	H = 35.28 *p* = 0.000	F = 19.88 *p* = 0.000	H = 34.56 *p* = 0.000	H = 36.67 *p* = 0.000	H = 36.66 *p* = 0.000	H = 35.73 *p* = 0.000	H = 15.79 *p* = 0.000	H = 33.89 *p* = 0.000
*Phacelia*	H = 36.65 *p* = 0.000	H = 36.63 *p* = 0.000	H = 36.47 *p* = 0.000	H = 35.49 *p* = 0.000	H = 34.78 *p* = 0.000	H = 36.62 *p* = 0.000	H = 36.65 *p* = 0.000	H = 36.62 *p* = 0.000	H = 36.64 *p* = 0.000	H = 30.30 *p* = 0.000
Buckwheat	H = 36.63 *p* = 0.000	H = 36.71 *p* = 0.000	H = 36.61 *p* = 0.000	H = 10.84 *p* = 0.013	H = 33.46 *p* = 0.000	H = 36.65 *p* = 0.000	H = 36.64 *p* = 0.000	H = 34.82 *p* = 0.000	H = 22.82 *p* = 0.000	H = 34.43 *p* = 0.000
Goldenrod	H = 36.61 *p* = 0.000	H = 36.68 *p* = 0.000	H = 33.08 *p* = 0.000	F = 22.06 *p* = 0.000	H = 34.30 *p* = 0.000	H = 36.65 *p* = 0.000	H = 36.71 *p* = 0.000	H = 36.63 *p* = 0.000	H = 31.78 *p* = 0.000	H = 33.06 *p* = 0.000

H—value of statistics for the Kruskal–Wallis test; F—value of Fisher’s test for ANOVA; *p*—probability value.

## Data Availability

Data contained within the article. At a justified request of the interested party, they may be made available by the corresponding author.
